# BCMA嵌合抗原受体T细胞治疗5例IgD型复发/难治多发性骨髓瘤的疗效和安全性分析

**DOI:** 10.3760/cma.j.issn.0253-2727.2023.12.012

**Published:** 2023-12

**Authors:** 雁春 贾, 小祥 王, 琬婷 强, 进 刘, 沛 郭, 静 卢, 晓强 范, 海燕 何, 鹃 杜

**Affiliations:** 海军军医大学第二附属医院血液病科，全军骨髓瘤与淋巴瘤疾病中心，上海 200003 Department of Hematology, Myeloma & Lymphoma Center, Second Affiliated Hospital of Navy Medical University, Shanghai 200003, China

IgD型多发性骨髓瘤（MM）是一种罕见亚型，发病年龄低、肿瘤负荷高、预后不良[1]。即使在新药时代，IgD型MM的治疗仍极具挑战，总生存（OS）期仅为18.5～36.5个月[1]–[3]，复发/难治MM（RRMM）患者预后更差。近年来，基于BCMA靶点的嵌合抗原受体T细胞（CAR-T细胞）治疗RRMM取得令人鼓舞的成果，国外已经有2种CAR-T细胞治疗产品获批上市[4]–[6]，我国在今年6月30日也批准了第一款CAR-T产品。但CAR-T是否能提高IgD型MM患者的疗效目前研究较少，本研究我们总结BCMA CAR-T治疗RRMM患者的临床试验（NCT03093168），将5例IgD型RRMM患者的疗效、预后及不良反应报道如下。

## 病例与方法

一、病例资料

回顾性分析2018年11月至2019年7月采用BCMA CAR-T治疗RRMM的临床试验（NCT03093168）中5例IgD型RRMM患者的临床资料。诊断符合国际骨髓瘤工作组（IMWG）诊断标准[7]。收集临床资料包括性别、年龄、Durie Salmon（DS）分期、国际预后分期系统（ISS分期）、修订的ISS（R-ISS分期）、美国东部协作组体能状况评分（ECOG评分）、骨髓浆细胞比例（BMPC％）、血清游离轻链（sFLC）、M蛋白、髓外侵犯情况、荧光原位杂交（FISH）结果、既往治疗方案及线数、移植情况、疗效、微小残留病（MRD）及生存情况等。高危细胞遗传学异常参照Mayo骨髓瘤分层及风险调整治疗（Mayo Stratification of Myeloma And Riskadapted Therapy，mSMART）分层系统判定。

二、治疗方案

5例患者均采用FC方案进行预处理，具体为：氟达拉滨30 mg·m^−2^·d^−1^，−4～−2 d；环磷酰胺300 mg·m^−2^·d^−1^，−4～−2 d。0 d输注鼠源抗BCMA CAR-T细胞9×10^6^/kg。CAR-T细胞输注后密切随访监测，直至疾病进展出组。

三、疗效评价

按照2016年IMWG疗效评价标准[8]，包括严格意义的完全缓解（sCR）、完全缓解（CR）、非常好的部分缓解（VGPR）、部分缓解（PR）、微小缓解（MR）、疾病稳定（SD）、疾病进展（PD）。总缓解率（ORR）定义为最佳疗效≥PR。OS期定义为从患者接受CAR-T细胞回输到任何原因引起死亡或末次随访的时间；无进展生存（PFS）期定义为从患者接受CAR-T细胞回输到观察到疾病进展或发生任何原因的死亡的时间。

四、不良反应

根据Lee等的标准进行细胞因子释放综合征（CRS）分级[9]，不良反应参考美国国家癌症研究所常见不良反应评价标准（4.03版）。CRS和不良反应的判断由临床医师独立进行。

## 结果

例1，男性，43岁。于2015年6月因“颈肩部疼痛”确诊为MM伴髓外侵犯（肋骨旁），IgD-λ型。在外院给予CBD（环磷酰胺+硼替佐米+地塞米松）方案诱导后行自体造血干细胞移植（auto-HSCT），达CR。移植后21个月PD，先后接受5线治疗。2018年11月疾病再次进展，入组CAR-T临床试验。给予Bi-RCD（克拉霉素、来那度胺、环磷酰胺、地塞米松）方案桥接治疗，疗效为PD。回输前评估MM合并髓外侵犯（肋骨旁、椎体旁），DS ⅢB期，ISS Ⅱ期，R-ISS Ⅱ期，ECOG 0分。2018年12月18日行CAR-T细胞回输，回输后CRS 2级，予托珠单抗联合甲泼尼龙干预好转。回输后28 d评估VGPR，66 d达到MRD阴性，112 d时达到sCR，随访至2023年6月25日疾病未进展。

例2，男性，68岁。于2014年7月因“腰背疼痛”确诊为MM（IgD-λ型）。先后接受6线治疗，既往治疗药物包括硼替佐米、卡非佐米、沙利度胺、来那度胺。2018年8月疾病再次进展，入组CAR-T临床试验。入组时评估DS ⅢB期，ISS Ⅲ期，R-ISS Ⅲ期，ECOG 2分；合并17p−（p53缺失）、1q21+、t（11;14），予达雷妥尤单抗+地塞米松桥接治疗达到VGPR疗效。2018年11月20日进行CAR-T细胞回输，最佳疗效达VGPR，MRD阴性。回输后12个月再次进展，给予挽救性治疗，于回输CAR-T后21个月死于PD。

例3，男性，70岁。于2017年11月因“血小板减少”就诊，诊断为MM（IgD-λ型）。先后予3线治疗，治疗药物包括硼替佐米、来那度胺，最佳疗效VGPR。2018年8月疾病再次进展，外周血浆细胞比例44％，为浆细胞白血病阶段，入组CAR-T临床实验。回输前给予桥接治疗，疗效为PD。回输前DS ⅢB期，ISS Ⅲ期，R-ISS Ⅲ期，ECOG 2分。合并1q21+、17p−。2018年11月16日回输CAR-T细胞，CRS 2级。回输后首次评估疗效为sCR MRD阴性，回输后4个月疾病进展，并于回输后6个月死于PD及多器官功能障碍综合征（MODS）。

例4，男性，37岁。于2014年9月因“恶心、呕吐伴体重下降”确诊为MM（IgD-λ型）。先后予7线治疗。2018年10月评估疾病再次进展，入组CAR-T临床试验。予V-DCEP方案（硼替佐米、地塞米松、环磷酰胺、依托泊苷、顺铂）桥接治疗，疗效为PD。回输前评估MM合并髓外侵犯（肝、脾），DS ⅢB期，ISS Ⅲ期，R-ISS Ⅲ期，ECOG 1分。2018年12月9日进行CAR-T细胞回输，细胞扩增不佳（最大为5 366拷贝数/µg），回输后14 d评估疗效为SD，28 d评估为PD，回输后25个月因PD死亡。

例5，女性，50岁。于2017年1月因“右眼球突出”就诊，确诊为MM伴髓外侵犯（右眼眶），IgD-λ型。先后予5线治疗，既往治疗药物包括硼替佐米、伊沙佐米、卡非佐米、沙利度胺、来那度胺、泊马度胺。DS ⅡA期，ISS Ⅱ期，R-ISS Ⅱ期，ECOG 3分。2019年6月疾病再次进展。2019年7月29日行CAR-T细胞回输，回输后检测CAR-T细胞扩增不佳（最大为8652拷贝数/µg），14 d评估为PD，并于39 d因PD及MODS死亡。

## 讨论及文献复习

IgD型MM是一种较为罕见的亚型，预后极差。1975年一项纳入133例IgD患者的研究显示中位OS期仅为13.7个月[10]。2011年Kim等[3]报告75例IgD患者中位OS期为18.5个月[3]。一项来自中国的研究显示IgD患者的中位PFS和OS期分别为9.5和20.5个月[11]。2021年，我中心回顾性分析了亚洲骨髓瘤工作网（AMN）的356例IgD患者，发现即使是在新药时代，IgD患者的中位OS期也仅有3年左右[1]。同年，欧洲血液和骨髓移植学会（EBMT）的研究显示，即使接受auto-HSCT，IgD患者的中位OS期也显著低于其他亚型患者（48.5个月对81.7个月）[12]。RRMM患者预后更差。

近年来，CAR-T疗法在治疗RRMM方面取得里程碑式的进展，一项Meta分析显示ORR为82％，CR率为36％[13]。Raje等[5]报道一种BCMA CAR-T疗法治疗RRMM患者的ORR为85％，中位PFS期为11.8个月，中位缓解持续时间（DOR）为10.9个月。在一项人源化抗BCMA CAR-T治疗RRMM的临床试验中，ORR为96.4％（27/28），疗效达到CR患者的MRD阴性率为93.7％[14]。另一项靶向G蛋白偶联受体C5家族亚型D（GPRC5D）的CAR-T治疗RRMM的Ⅰ期临床试验中，ORR达到71％，DOR为7.8个月[15]。Xia等[16]最近报道33例接受抗GPRC5D CAR-T治疗的RRMM患者的结果，中位随访5.2个月，ORR达到91％，sCR率33％。由于IgD亚型的罕见性，CAR-T疗法在IgD型RRMM中的有效性和安全性少有报道。

本研究的5例IgD型 RRMM患者，既往曾接受蛋白酶体抑制剂、免疫调节剂、CD38单抗等多线治疗，2例既往行auto-HSCT，3例ISS及R-ISS为Ⅲ期，3例合并髓外侵犯，整体极高危、预后极差。接受BCMA CAR-T治疗后3例有效，2例达sCR，治疗有效的患者均获得MRD阴性。在未予其他药物治疗前提下，中位PFS期为4.5个月，中位OS期为21.2个月，其中1例患者持续sCR达到59个月。与治疗有效患者相比，无效患者（例4和例5）CAR-T细胞扩增不佳。结合现有数据及文献，我们认为CAR-T扩增不良主要与既往多线治疗有关，其他可能因素还包括T细胞功能低下、骨髓瘤微环境影响等[17]。提示在未来的研究中可在疾病早期阶段进行T细胞采集，对高危患者尝试将CAR-T治疗线数前移，以期获得更好疗效。

CRS是CAR-T治疗的主要不良反应。本研究中，2例患者在接受CAR-T细胞回输后出现2级CRS，均接受糖皮质激素治疗，其中1例同时接受托珠单抗处理后症状好转；所有患者未出现免疫效应细胞相关神经毒性综合征（ICANS）。血液学毒性仍是最常见不良反应，包括各类血细胞的减少，这与既往报道类似[4]。非血液学不良反应中，低钾血症、低白蛋白血症发生率为100％，低钙血症发生率为80％，多为1～2级，接受对症处理后好转，本研究CAR-T治疗的患者总体不良反应可控，经对症处理均好转。

2021年，Chen等[18]对接受BCMA-CD19 CAR-T或CD19 CAR-T的7例IgD型RRMM患者分析，其中6例达sCR，1例合并髓外侵犯的患者疗效为MR，ORR为85.7％。本组病例疗效低于Chen等[18]的研究。这可能与本研究中高危细胞遗传学异常比例较高、高肿瘤负荷、髓外侵犯、治疗线数多等相关，且Chen等报道使用BCMA和CD19的双靶点联合CAR-T疗法也可能更利于疗效的提高。

IgD型MM患者疗效评估大多参比轻链型患者的评估体系，导致疗效评估不够精准[19]。因此，我中心2022年基于大样本研究，整合IgD定量的疗效评估标准，对IgD型MM患者的疗效评估进行优化[19]。在本研究应用了此评估体系，疗效和预后得精准度得到提升。
